# The 3D revolution: organoids and spheroids reshape parasitology research

**DOI:** 10.1017/S0031182025101273

**Published:** 2025-09

**Authors:** Cinzia Cantacessi

**Affiliations:** Department of Veterinary Medicine, University of Cambridge, Cambridge, UK

## Abstract

**Figure:**
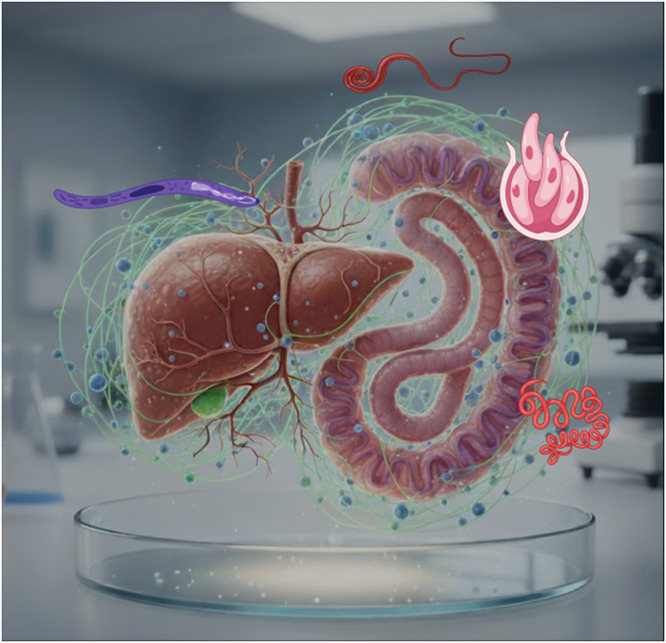

Fundamental studies of parasitic diseases of humans and animals, and of host-parasite interactions, have historically been hampered by two key challenges, i.e. the complex life cycles of the pathogens, that often involve multiple vertebrate and invertebrate hosts, and an often unavoidable reliance on whole-animal models. The latter, while necessary, is restrictive, expensive, and ethically burdensome, while traditional 2D cell cultures often fail to replicate the nuanced tissue environments that parasites require for full development. The articles compiled in this Virtual Collection, stemming from the groundbreaking symposium ‘Miniature Worlds: Organoid Research in Parasitology’ (held in Cambridge on 8^th^ November 2024 with the support of *Parasitology* and the Infection & Immunity Theme of the School of Biological Sciences, Cambridge University), signal a profound paradigm shift: the integration of 3D cell culture technologies – specifically organoids and spheroids – towards antiparasitic drug discovery and fundamental research in host-pathogen interactions. These models provide biologically relevant, accessible, and scalable platforms that mimic native tissues, allowing researchers to observe previously unobservable aspects of parasitic life cycles.

The immediate implications for animal health are significant. Two articles detail breakthroughs in tackling coccidiosis, an economically devastating disease in the poultry industry caused by protozoans of the genus *Eimeria*. The review article by Chan et al. introduces novel organoids and *ex vivo* models, laying the groundwork for more advanced studies in the biology of these parasites. Building on this, the cultivation of the complete endogenous life cycle of *Eimeria tenella* in chicken intestinal organoids (Teng et al.) marks a critical technical milestone. For the first time, researchers can isolate and study the full parasitic phase of development outside of a living chicken, drastically reducing the need for costly animal trials and accelerating the screening of new anti-coccidial compounds.

Extending beyond apicomplexan parasites, the utility of this technology for studies of host-nematode interactions is highlighted by the review by Perez et al. Nematodes are large, complex organisms, and their life cycles are heavily dependent on tissue architecture and specific host signalling pathways. Organoids provide the complex epithelial surface and cellular communication necessary to understand how these parasites invade, migrate, and establish chronic infections, offering new targets for drug intervention against diseases like ascariasis and hookworm.

The development of a 3D spheroid model for *Plasmodium falciparum* represents an exciting application of this technology to parasites of human health significance. The research article by Caygill et al. describes an accessible 3D HepG2-C3A liver spheroid model capable of supporting the complete intrahepatocytic life cycle of this parasite; the latter stage is a crucial but often inaccessible bottleneck in malaria research. The model more closely resembles the *in vivo* environment of the hepatocyte layer, thus allowing for accurate observation and, critically, high-throughput screening against the hypnozoite (dormant) stages and the highly proliferative schizont stage. This accessibility is paramount, as the development of novel drugs targeting the liver stage is a global priority for achieving malaria eradication.

These four reports collectively underscore the transformative power of bioengineering in parasitology. Organoids and spheroids offer unparalleled opportunities to decouple the pathogen from the host, standardize experimentation, and increase throughput in the search for urgently needed antiparasitic drugs and vaccines.

We are entering an era where drug efficacy can be assessed in tissue-like structures before moving to animal models, thus optimizing the pipeline and adhering to ethical considerations. The challenge now lies in expanding this platform – adapting the 3D models to replicate the tissue tropism of other major parasites, such as hookworms. The successful cultivation of complex life cycles in these miniature organs is not only a scientific achievement, but a catalyst for the next generation of breakthroughs in disease control.

